# Cardiomyopathies: The Role of Non-Coding RNAs

**DOI:** 10.3390/ncrna10060053

**Published:** 2024-10-23

**Authors:** Nicole Carabetta, Chiara Siracusa, Isabella Leo, Giuseppe Panuccio, Antonio Strangio, Jolanda Sabatino, Daniele Torella, Salvatore De Rosa

**Affiliations:** 1 Department of Medical and Surgical Sciences, Magna Graecia University, 88100 Catanzaro, Italy; nicole.carabetta95@gmail.com (N.C.); siracusachiara30@gmail.com (C.S.); 2 Department of Experimental and Clinical Medicine, Magna Graecia University, 88100 Catanzaro, Italy; isabella.leo@unicz.it (I.L.); panuccio@unicz.it (G.P.); antonio.strangio@unicz.it (A.S.); sabatino@unicz.it (J.S.); dtorella@unicz.it (D.T.); 3 Department of Cardiology, Angiology and Intensive Care Medicine, Deutsches Herzzentrum der Charité Berlin, 12200 Berlin, Germany

**Keywords:** non-coding RNAs, micro-RNAs, long non-coding RNAs, cardiomyopathies

## Abstract

Cardiomyopathies are the structural and functional disorders of the myocardium. Etiopathogenesis is complex and involves an interplay of genetic, environmental, and lifestyle factors eventually leading to myocardial abnormalities. It is known that non-coding (Nc) RNAs, including micro (mi)-RNAs and long non-coding (lnc) RNAs, play a crucial role in regulating gene expression. Several studies have explored the role of miRNAs in the development of various pathologies, including heart diseases. In this review, we analyzed various patterns of ncRNAs expressed in the most common cardiomyopathies: dilated cardiomyopathy, hypertrophic cardiomyopathy and arrhythmogenic cardiomyopathy. Understanding the role of different ncRNAs implicated in cardiomyopathic processes may contribute to the identification of potential therapeutic targets and novel risk stratification models based on gene expression. The analysis of ncRNAs may also be helpful to unveil the molecular mechanisms subtended to these diseases.

## 1. Introduction

Cardiomyopathies are defined by the recently published European guidelines as “a myocardial disorder in which the heart muscle is structurally and functionally abnormal, in the absence of coronary artery disease (CAD), hypertension, valvular disease, and congenital heart disease (CHD) sufficient to cause the observed myocardial abnormality” [1,2].

The guidelines also recommend that these should be classified according to distinct functional and anatomical characteristics, using a phenotypic approach; in detail, this classification includes dilated cardiomyopathy (DCM), hypertrophic cardiomyopathy (HCM), non-dilated left ventricular cardiomyopathy (NDLVC) and arrhythmogenic ventricular cardiomyopathy (ACM) [2]. Each of these phenotypes are then further sub-classified into acquired and inherited variants [3].

However, an exact phenotypical classification may be challenging in real word scenarios and often requires the use of novel advanced biomarkers that already have been proven to improve the diagnostic and prognostic evaluation of cardiovascular diseases [4,5,6,7,8].

Genetic testing is part of the diagnostic pathway of these patients; however, cardiomyopathies are characterized by a significant genetic heterogeneity, with several different variants of different genes potentially resulting in the same phenotypical expression. In addition, incomplete penetrance and variable expressivity further complicate the interpretation of genetic findings. In this regard, investigating gene expression at the post-transcriptional level may be helpful to sort out all the pieces of the puzzle.

These cardiomyopathies are associated with a large variety of abnormalities in sarcomeres, mechano-transduction and excitation–contraction coupling genes [9,10,11].

**Figure 1:**
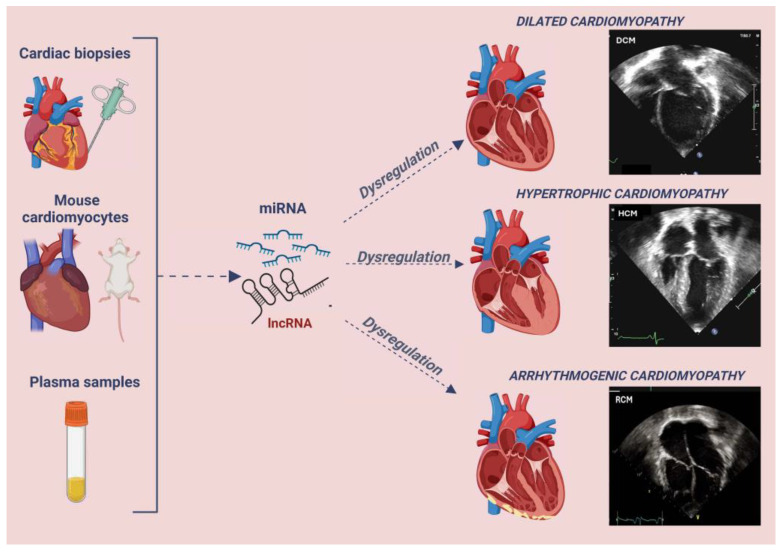
Non-Coding RNAs and their sources in the analyzed studies on cardiomyopathies. Figure created with Biorender; echocardiographic images are part of our archive.

Although a small percentage of these patients have specific genetic mutations, there are other mechanisms that are possibly responsible for the evolution of these diseases; among these, non-coding RNA (ncRNA), especially long non-coding RNA (lncRNA) and micro-RNA (miRNA), has already been demonstrated to have a role in different conditions and may also be relevant in this context (Figure 1) [12].

This is particularly relevant if we recognize that even if certain mutations are implicated in specific pathologies, mutations in the same gene may result in different clinical phenotypes; the interaction of incomplete penetrance, mutational heterogeneity and other factors can complicate clinical scenarios and diagnostic pathways [13].

## 2. Non-Coding RNAs

NcRNAs constitute a broad category of RNA molecules that, despite not translating into proteins, play a crucial role in regulating gene expression at various levels [14]; they participate in numerous cellular processes, including development, differentiation and cellular proliferation [15].

Based on their length, ncRNAs can be classified into two main categories: miRNAs and lncRNAs; in addition to linear ncRNAs, there is another class known as circular RNAs (circRNAs), which have a covalently closed loop structure [16].

miRNAs are small non-coding RNA molecules, approximately 22 nucleotides long, that regulate post-transcriptional gene expression by modulating mRNA stability and translation. Specifically, miRNAs bind complementarily to the 3′ untranslated region of target mRNAs, leading to gene expression repression or mRNA degradation [17].

In recent years, numerous studies have explored the role of miRNAs in the development of various pathologies, including heart diseases such as ischemia and cardiomyopathies [18].

MiRNAs also interact with another family of regulatory RNAs, namely lncRNAs; these are a highly heterogeneous class of RNA molecules, approximately 200 nucleotides long, which regulate post-transcriptional expression by binding to miRNAs and preventing them from binding to target messenger RNAs (mRNAs), leading to gene expression inhibition [19,20]. 

CircRNAs are a class of non-coding RNA characterized by a highly stable covalently closed loop structure [21]. CircRNAs can modulate gene expression through various mechanisms, including interaction with RNA-binding proteins or binding to miRNAs, thereby preventing them from interacting with their target mRNAs [22,23]. In cardiovascular diseases, circRNAs have emerged as important regulators of key cellular processes, including apoptosis, fibrosis and cellular inflammation. The deep sequencing of cardiac tissue has revealed the abundance, evolution, conservation and stage-specific differentiation of circRNA expressed in the heart [24]. Alterations in circRNA expression have been observed in conditions such as myocardial infarction, heart failure and cardiac hypertrophy. In particular, heart-related circRNA (HRCR) appears to be repressed in hypertrophic hearts and in heart failure [25]. The forced expression of HRCR attenuated cardiomyocyte hypertrophy in vitro and in vivo, suggesting that circRNAs may be involved in the progression and maintenance of cardiovascular diseases. The circRNA CDR1as increases infarct size and cardiomyocyte apoptosis [25]. The analysis of human and mouse circRNA data revealed that 15% of circRNAs are precisely conserved splice sites in orthologous genes, and 10% of circRNAs are conserved in human, mouse and rat hearts [26]. The study of circRNAs conserved between the two species is of great importance for the discovery of potential biomarkers for early diagnosis, as well as promising therapeutic targets for these conditions (circRNAs expressed in human peripheral blood are associated with human aging phenotypes, cellular senescence and mouse lifespan).

**Figure 2:**
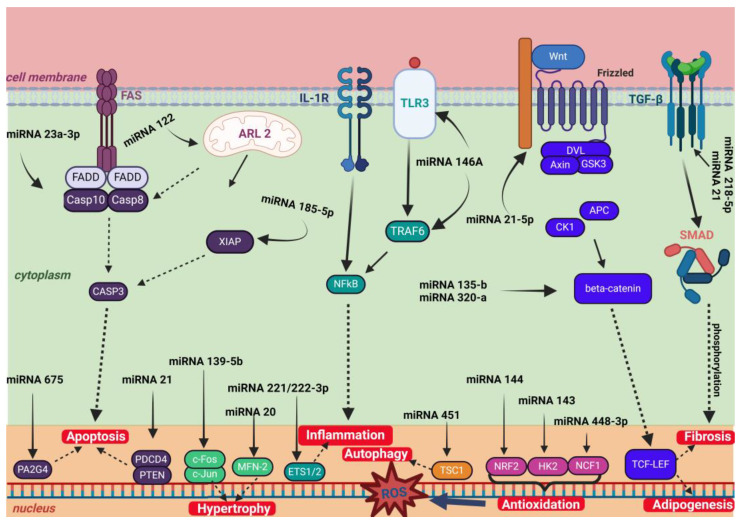
Mechanisms of action of some well-known miRNAs in cardiomyopathies. Figure created with Biorender.

ncRNAs have demonstrated fundamental roles in regulating gene expression and contribute, through various mechanisms, to the pathogenesis of cardiomyopathies [27] Understanding how different types of ncRNAs—including miRNAs and lncRNAs—influence the development of cardiomyopathies could pave the way for new diagnostic and therapeutic strategies for managing cardiovascular diseases (Figure 2).

## 3. Dilated Cardiomyopathy

Of all cardiomyopathies, DCM is the most prevalent, and is characterized by left ventricle dilatation associated with a global systolic dysfunction [28,29,30,31,32]. Patients with DCM may remain asymptomatic or pauci-symptomatic for years before it eventually progresses to heart failure, thromboembolism and, in some cases, sudden cardiac death [33].

Pathogenic gene variants, toxins, infections, auto-immunity, tachyarrhythmias and storage diseases are all causes of DCM. In genetic forms of DCM, mutations occur more in cytoskeletal and sarcomeric proteins [34,35,36,37]. Conversely, DCM can also represents the cardiac expression of a systemic genetic disease involving the neuromuscular system, such as Emery–Dreifuss, Becker or Duchenne muscular dystrophy [38].

The inheritance of this condition is in most cases autosomal-dominant, and in a smaller number of cases, autosomal recessive or x-linked [30,32,39,40]. Over 50 genes have been linked to DCM, in particular, the most involved are myosin heavy chain 7 (MYH7), dystrophin gene (DMD), desmoplakin (DSP), vinculin (VCL), desmin (DES), sodium voltage-gated channel alpha subunit 5 (SCN5A), actin alpha cardiac muscle 1 (ACTC1), nexilin F-actin binding protein (NEXN), troponin T (TNNT2), RNA-binding motif protein 20 (RBM20), phospholamban (PLN), Lamin A/C (LMNA), tropomyosin 1 (TPM1), troponin C1 (TNNC1), BAG family molecular chaperone regulator 3 (BAG3) and titin (TTN) [10,41,42,43,44,45].

However, due to the low frequency and high heterogeneity of these mutations, some of the subjects affected by DCM do not have characteristic variants [44]. For this reason, the role of other portions of the genome, such as lncRNA and miRNA, in the pathogenesis of DCM must be underlined.

For example, a possible variant of TNNI3 interacting kinase (a gene that regulates cardiac contraction) has been highlighted. This variant manifests itself through a loss of gene function, as a result of structural alterations in chromatin and/or disruption in regulatory regions; all this leads to haploinsufficiency and therefore to a myocardial structural disorder [46]. In addition, several mutations have been identified in the non-coding regions of the FLNC gene, which may lead to DCM [46].

Additionally, ncRNA variations could change the natural history of the disease, differentiating an early stage dilated cardiomyopathy from a more advanced form. According to a recent study by Zhang et al., a long ncRNA SNHG9 (small nucleolar RNA host gene 9) could be used as a biomarker for dilated cardiomyopathy, because it can distinguish DCM stage III New York Heart Association (NYHA) from stage I or II NYHA and from healthy controls [47].

In another recent study, it was seen that the lncRNA CHKB-DT expression was marked as downregulated in DCM, and being a lncRNA related to energy metabolism and mitochondrial dysfunction, CHBK-DT may be a potential therapeutic target for DCM [48]. In addition, the lncRNA DRCT was overexpressed in normal cardiac tissues while it was downregulated in patients with DCM; its mechanism of action seems to involve mitochondrial dysfunction; in particular, DCRT inhibited the exon skipping of NDUFS2 (NADH dehydrogenase ubiquinone iron-sulfur protein 2) by binding to PTBP1 (polypyrimidine tract binding protein 1) [49].

A fibroblast-associated lncRNA (CFIRL) seems to be upregulated in patients with heart failure and DCM, as it promotes fibroblast differentiation and improves angiotensin II-related differentiation to myofibroblast [50]. In contrast, lnc-RNA H19 associated with miRNA-675 contributes to the induction of cardiomyocyte apoptosis; by targeting PA2G4, its downregulation in the myocardium of DCM rats improved left ventricular function (Table 1) [51].

Furthermore, a strong correlation between two intronic susceptibility loci in HSPB7 and FRMD4B, namely rs1739843 and rs6787362, and progressive heart failure has been demonstrated in the literature [52].

MiRNAs have crucial functions in the pathophysiology of cardiac disease [53]; in particular, mounting evidence has shown a significant role of inflammation in DCM, and in this context, miRNA, as important inflammatory regulators, plays a key role [54,55,56].

Both MiRNA-155 and miRNA-133a levels were increased in endomyocardial biopsies from patients who had inflammatory DCM; higher levels of miRNA-133a, which plays a role in the modulation and reduction of inflammation, have also been linked to reduced fibrosis, preserved LV systolic function and better cardiac outcomes [57].

In another study, it appears that in DCM, the down-regulation of miRNA 451a may play a role in inflammation process by targeting the transcription factor Myc and promoting the activation of CD4+ T cells [58].

MiRNA 486 and MiRNA-146a regulate inflammation and cell viability; specifically, it has been demonstrated that patients with heart failure have higher levels of circulating exosomal miR-146a, as part of an inflammatory process involving factors such as tumor necrosis factor-alpha (TNF-α) and granulocyte–macrophage colony-stimulating factor (GM-CSF) [59].

On the other hand, results from real-time PCR indicated that the expression of miRNA 181b in patients affected by HF was lower than in healthy ones; this is due to the fact that interleukin-1β (IL-1β), interleukin-6 (IL-6) and TNF-α inhibit this miRNA [60].

In vitro studies of transgenic mice that overexpress miRNA-30c have shown the possible involvement of this miRNA in the development of DCM, as these mice developed severely dilated cardiomyopathy after six weeks. In these mice, mitochondrial oxidative phosphorylation (OXPHOS) complexes III and IV were downregulated, which resulted in a coexistence of mitochondrial dysfunction [61]. Likewise, DCM was observed in transgenic mice overexpressing miRNA-7, associated with a reduction in NDUFA9, a component of NADH dehydrogenase complexes [62].

In humans, there was a strong correlation between impaired cardiac function, as measured by ejection fraction and NT-proBNP levels, and the elevation of circulating exosomal miRNA-194, miRNA-29a, miRNA 122, and miRNA 423-5p [63,64,65,66].

Oxidative stress also plays a role in the development of cardiac dysfunction; in fact, some studies have demonstrated that the downregulation of miR-448-3p results in the increased expression of the NCF1 gene and p47phox protein, along with a significant rise in the generation of reactive oxygen species (ROS); this leads to tissue myocardial damage and cardiac dysfunction [67].

Furthermore, in mouse cardiomyocytes, the transgenic overexpression of miRNA-143/145, which promoted a reductive redox shift through several mechanisms, produced a condition resembling dilated cardiomyopathy [68].

Other studies have investigated a possible involvement of miRNAs-221/222 in the development of heart failure, as they determine a significative reduction in autophagy, a fundamental mechanism of homeostasis, by downregulating P27 and activating mTOR [69,70].

However, in humans, the upregulation of miRNA-22 in CMD patients can set off a complicated cascade of events involving not only mTOR but also PP2Cm, which catalyzes branched chain amino acids (BCAAs); consequently, a reduction in PP2Cm levels promotes the buildup of BCAAs with proteostasis loss and inflammasome activation [71].

The individual inhibition of other miRNA that are responsible for cell survival, such as miRNA-1, miRNA-29c, miRNA-30c, miRNA-30d, miRNA-149, miRNA-486 and miRNA-499, promotes apoptosis, according to in vitro experiments performed on neonatal cardiomyocytes, and that process may be a factor in the loss of cardiac cells and heart failure [72].

Fibrosis is a key mechanism in the progression of DCM, and several miRNA are involved in this process, like miRNA-208, which is connected to an increase in myocardial collagen volume and worse clinical outcomes in DCM patients, and MiRNA-218-5p, which stimulates TGF-β and promotes a profibrotic effect [73,74].

In animal models, it has been reported that during adverse remodeling there is a downregulation of miRNA-26, miRNA-29, miRNA-30 or miRNA-133a and an increase in miRNA-21; these changes lead to an overexpression of profibrotic genes, contributing to increased collagen production and myocardial fibrosis [75,76,77]. Likewise, miRNA-33 has a role in cardiac remodeling and its expression may be connected to enhanced hemodynamic parameters in DCM patients [78].

Among circulating MiRNA that contribute to DCM, we have to mention miRNA-142-3p and miR-548 family, as their downregulation has been documented in patients with DCM and heart failure [79,80]. Additionally, the upregulation of some miRNA (miR-3135b, miR-3908, miR-5571-5p and exo-miR-92b-5p) may be used as DCM diagnostic biomarkers [81,82]. In contrast, the expression levels of miRNA 15-b-5p and miRNA-106a-5p may be helpful in differentiating an ischemic from an non-ischemic dilated cardiomyopathy [83].

A cellular microRNA let-7i, which regulates the expression of Toll-like receptor (TLR) 4, is implicated in the progression of DCM, determining worse clinical outcomes in these patients [84].

Beyond diagnosis, some miRNAs have a potential role in predicting the prognosis of subjects with DCM: the increased expression of miRNA185 is associated with a better prognosis, while miR-338-3p may be a predictive biomarker of adverse cardiovascular events in patients with dilated cardiomyopathy [85,86].

In a recent study that evaluated the cardiac biopsies of patients with virus-negative inflammatory DCM, the dysregulation of six inflammation-correlated miRNAs (miRNA-1, miRNA-23, miRNA-142-5p, miRNA-155, miRNA-193 and miRNA-195) has been identified as part of a cardiac inflammatory process and thus as a possible therapeutic target (Table 2 and Table 3) [87].

Finally, the following plasma exosomes of DCM patients with HF were found to be dysregulated: hsa-miRNA-3138_L-5R+2, which controls cardiac mitochondrial activity; hsa-miRNA-1304-3p_1ss13CA, which is implicated in inflammation and oxidative stress; and hsa-miRNA-10a-5p_R-1, which is linked to cardiac fibrosis via the TGF-β/Smad3 signaling pathway [88].

Therefore, several miRNAs are implicated in DCM pathogenesis; in particular, it has been shown that a dysregulation of miRNA-mRNA networks may promote changes at the level of the ECM with its accumulation, cardiac fibrosis, hypertrophy, inflammation, oxidative stress, angiogenesis, and cell death [89].

## 4. Hypertrophic Cardiomyopathy

Hypertrophic cardiomyopathy (HCM) is described as the presence of an elevated wall thickness (in the presence or absence of right ventricular hypertrophy) or mass that cannot be fully attributed to aberrant loading conditions [1].

The most common symptoms of HCM are palpitations, dyspnea, syncope, chest discomfort, heart failure, and arrythmias, which are similar and in overlap with the clinical presentation of other cardiomyopathies [13,90].

More than 20 genes are implicated in the pathogenesis of HCM; these genes mostly impact the sarcomere but other components, such as proteins of the Z-disk or intracellular calcium modulators, may be involved. MYH7 and the myosin-binding protein C3 (MYBPC3) are the two primary genes in which mutations occur in about 80% of cases; in 1–5% of cases, the following additional genes are involved in the pathophysiology of HCM: troponin I (TNNI3), TNNT2, myosin light chain 2 (MYL2), tropomyosin 1(TPM1), actin alpha cardiac muscle 1 (ACTC1), and Myosin Light Chain 3 (MYL3) [13,35,37,46,91].

However, in about 50% of individuals with HCM, genetic tests fail to identify a precise causal mutation [92]; the explanation could be the presence of mutations in non-coding regions, which are unable to be identified by genomic techniques. This could be the result of pathogenic mutations sited within the introns; in particular, several intronic variants have been found in the VCL, PRKAG2, and TTN genes [93]. These variants may trigger a frameshift and a stop codon, as in the case of intronic variants (c.1224-52G>A, c.1224-80G>A, c.1224-21A>G, and c.906-36G>A) in MYBPC3 [94].

LncRNAs are involved in the pathogenesis of HCM through the modulation of multiple pathogenetic pathways.

Recently, the function of lncRNA ADAMTS9 antisense RNA 1 (ADAMTS9-AS1) in HCM-induced cardiomyocyte hypertrophy has been demonstrated: its overexpression inhibited cardiomyocyte hypertrophy and decreased levels of ANP and BNP [95]. Furthermore, it has been seen that lncRNA–MIAT mediated the expression of miRNA29a-3p, which in turn is implicated in the control of fibrosis in HCM [96].

Another LncRNA “LncRNAh19” seems to act as a negative regulator of hypertrophy by targeting CaMKIIδ through miR-675 and its silencing or variants promote cardiac hypertrophy. It was discovered that patients with HCM end-stage had lower levels of lncRNA CAIF compared to controls; as a result, this lncRNA could be utilized as a prognostic and diagnostic marker [97,98].

Finally, the downregulation of the immune-related lncRNA–mRNA pair, MIR210HG–BPIFC, is related to the pathogenesis of this disease, involving the infiltration of naïve CD4+ T cells and CD8+ T cells in the myocardial cells of HCM patients (Table 4) [99].

miRNAs are also involved in HCM pathogenesis; it has been shown that three circulating miRNAs were upregulated in HCM patients compared to controls: miRNA-27a, miRNA-29a, and miRNA-199a-5p. They are correlated to cardiac remodeling, hypertrophy, and fibrosis [100]. In another study, in patients with mild to moderate HCM, the suppression of miRNA-199a-3p may provide therapeutic benefits [101].

Fang et al. identified 14 upregulated circulating miRNAs in HCM patients (miRNA-18a-5p, miRNA-146a-5p, miRNA-30d-5p, miRNA-17-5p, miRNA-200a-3p, miRNA-19b-3p, miRNA-21-5p, miRNA-193-5p, miRNA-10b-5p, miRNA-15a-5p, miRNA-192-5p, miRNA-296-5p, miRNA-29a-3p, and miRNA-133a-3p); these miRNAs contribute to cardiac fibrosis through a variety of pathways, including the control of TGF-β/CTGF signaling cascade, ECM proteins, fibroblasts, and epithelial-to-mesenchymal transition. Furthermore, the correlation of these miRNAs with cardiac fibrosis measured with cardiac magnetic resonance post-contrast T1 mapping values has been described [102].

Circulating miRNAs such as miRNA-181a-5p, -181c-5p, -193b-3p, -301a-3p, and -328-3p could stratify patients with HCM. In a recent study, they were significantly upregulated in clinical and subclinical HCM with early phenotypic manifestations (identified on echocardiogram and electrocardiogram) compared to subclinical HCM without early phenotypic manifestations [103].

It also appears that microRNAs may be involved in the prognosis of the patients with HCM; in fact, in subjects with end-stage HCM and heart failure there is an upregulation of some miRNAs, such as miRNA-1-3p, miRNA-23a-3p, miRNA-23b-3p, miRNA-24-3p, miRNA-29b-3p, miRNA-30d-5p, miRNA-125a-5p, miRNA-126-3p, miRNA-133a-3p, miRNA-143-3p, miRNA-145-5p, miRNA-193b-3p, miRNA-197-3p, miRNA-331-3p, miRNA-342-3p, miRNA-361-5p, miRNA-365-3p, miRNA-455-3p, miRNA-1975-3p, and miRNA-1978, compared to control groups [104,105].

Moreover, in HCM patients with MYBC3 mutation, there is a dysregulation of miR-10b and miR-10b* (downregulated) and miR-184, miR-497, miR-204, miR-34b*, and miR-222* (upregulated); by analyzing in silico models, mRNAs have been identified as targets of these microRNAs, which are implicated in ventricular hypertrophy and β-adrenergic pathway [106]. In another study conducted on patients with MYBC3 mutation, there was a considerable upregulation of miRNA-208b-3p associated with cardiac hypertrophy through the Wnt signaling pathway [107].

In contrast, in carriers of pathogenic/probably pathogenic (P/LP) variants in the MYH7 gene, an increase in miR-499a-5p was found compared to healthy controls or compared to carriers of mutations in the MYBPC3 gene [108].

MicroRNAs appear to be crucial regulators of cardiac remodeling; in this context, Song, L., et al. revealed that a decrease in miRNA-451 in cardiac cells promoted autophagosome formation by targeting TSC1 and might contribute to the development of HCM [109]. Likewise the downregulation of micro-RNA-139-5p could promote cardiac hypertrophy by targeting the transcription factor C-JUN [110]. Additionally, in another study, the upregulation of miRNA-20 caused the reduced expression of the factor *MFN2*, resulting in cardiac hypertrophy [111].

Finally, microRNAs could be used as prognostic biomarker; for example, miRNA-221 seems to be correlated with myocardial fibrosis and hypertrophy, and it is upregulated in patients with hypertrophic obstructive cardiomyopathy (HOCM) [112].

Recently, in human peripheral blood mononuclear cells (PBMCs) three potential biomarker miRNAs (miR-1, miR-98, miR-924) were screened using multiple machine learning algorithms that could be used for HCM detection [113]; via this method, another study determined that miRNA-124-3p may have a role in the pathophysiology of HCM through the regulation of the RhoA signaling pathway (Table 5 and Table 6) [114].

Transcriptomic analyses have just highlighted a possible role of an immune-related lncRNA–mRNA pair (*MIR210HG–BPIFC*) involved in the development of HCM through the infiltration of naïve CD4+ and CD8+ lymphocytes; these results suggest the use of new biomarkers as well as new therapeutic strategies in HCM patients [99].

## 5. Arrhythmogenic Cardiomyopathy

Arrhythmogenic cardiomyopathy (ACM) is a genetically determined cardiomyopathy characterized by the fibro-fatty infiltration of the myocardium. Initially, described as a condition affecting only the right ventricle, it has more recently been described in the left ventricle, either in association with the right or in an isolated “left dominant” form [115]. Subjects with ACM are clinically at risk of SCD, ventricular dysfunction and potentially fatal ventricular arrhythmias [116].

ACM is commonly inherited in an autosomal dominant pattern with incomplete penetrance and variable expressivity involving, in most cases, the pathological mutations of cardiac desmosomal components [117]. Desmoplakin (DSP), plakophilin (PKP2), desmocollin (DSC2), and desmoglein (DSG2) are the most often mutated proteins in this disease, but mutations in genes involving non-desmosomal components (in particular cytoskeletal components and ion channels) have also been described; these involve desmin (DES), phospholamban (PLN), transforming growth factor (TGF-β3), filamin C (FLNC), cardiac sodium channel (SCN5A), and transmembrane protein-43 (TMEM43) [118,119].

In a recent study, a potential lncRNA–miRNA–mRNA regulatory network seems to be implicated in the pathogenesis of ACM, in particular, in two lncRNAs, XIST and LINC00173, in addition to four mRNAs, FBN1, COL1A1, COL5A1, and BGN; recognized through the competitive endogenous RNA (ceRNA) network, these may be useful for diagnosis and could be a target for ACM treatment in the future [120].

The analysis of miRNA expression in plasma samples taken from ACM and controls demonstrated that miRNA-320a is substantially less expressed in patients with ACM, potentially representing a disease biomarker [121]. Another study confirmed the possible role of miRNA-185-5p, which was significantly upregulated in ACM patients, compared to controls [122].

MicroRNAs are also able to distinguish between ACM and other cardiomyopathies; in fact it has been demonstrated that miR-122-5p, miR-133a-3p, miR-133b, miR-142-3p, miR-182-5p, and miR-183-5p were dysregulated compared to healthy controls, non-affected family members of AC probands with a desmosomal pathogenic variant, and patients with other cardiac disease such as HCM, DCM, and myocarditis [123].

Twenty-four dysregulated miRNA were found in the heart tissue of transplant patients with ACM, twelve of which were upregulated (miRNA-21-3p, miRNA-21-5p, miRNA-34a-5p, miRNA-212-3p, miRNA-216a, miRNA-584-3p, miRNA-1251, miRNA-3621, miRNA-3674, miRNA-3692-3p, miRNA-4286, and miRNA-4301) and twelve of which were downregulated (miRNA-135b, miRNA-138-5p, miRNA-193b-3p, miRNA-302b-3p, miRNA-302c-3p, miRNA-338-3p, miRNA-451a, miRNA-491-3p, miRNA-575, miRNA-3529-5P, miRNA-4254, and miRNA-4643). Specifically, the two most severely dysregulated components were MiRNA-21-5p and miRNA-135b, implicated in Wnt and Hippo signaling pathways, and were thus involved in fibrosis and fibrofatty infiltration [124,125].

Moreover, in ACM cardiac stromal cells, miRNA-29b-3p was upregulated compared to controls [126].

Finally, five microRNAs (hsa-miRNA-1-3p, hsa-miRNA-21-5p, hsa-miRNA-122-5p, hsa-miRNA-206, and hsa-miRNA-3679-5p) potentially related to the pathogenesis of ACM were also found to be differentially expressed between patients with ARVC and patients with post-infarction ventricular tachycardia undergoing ventricular ablation [127].

MiRNAs also correlate with the prognosis of ACM patients. Yamada et al. found higher plasma levels of miRNA-144-3p, 145-5p, 185-5p, and 494 in patients with ACM complicated with ventricular arrhythmias [128] (Table 7).

## 6. Conclusions

Due to evidence of their possible involvement in pathophysiology, non-coding RNA, in particular lncRNAs and miRNAs, have garnered enormous attention as promising diagnostic and prognostic biomarkers in cardiovascular disorders. In fact, these may not only help in the early identification of phenotype-negative patients at higher risk of expressing the disease but also in distinguishing among different cardiomyopathies with similar phenotypical and clinical presentation. In addition, several studies already highlighted the potential role of several ncRNAs as marker for risk stratification in cardiomyopathies and as potential target for novel customized therapeutic strategies. An exact comprehension of the role of ncRNAs in this context might help in unveiling biological processes underlying disease’s progression. Further research is certainly needed to justify their routine use, and there is still a long way to go before a miRNA-based approach is established in clinical practice; however, this promising evidence maps out potential novel pathways to be followed in the future.

## Figures and Tables

**Table 1:** lnc-RNAs in dilated cardiomyopathy.

lncRNAs	Quantitative Effect	Source	Notes
lncRNA SNHG9	Upregulated	Plasma sample of patients with heart failure	It is negatively associated with heart function [47].
lncRNA CFIRL	Upregulated	Hearts of patients with dilated cardiomyopathy (DCM)	It promotes fibroblast differentiation and improves angiotensin II-related differentiation to myofibroblast [50].
lnc-RNA H19	Upregulated	Mouse models of DCM	Contributes to the induction of cardiomyocyte apoptosis by targeting PA2G4; its downregulation improves the left ventricular function [51].
lncRNA CHKB-DT	Downregulated	Mouse models of DCM	It is related to energy metabolism and mitochondrial dysfunction. It inhibits the exon skipping of NDUFS2 (NADH dehydrogenase ubiquinone iron–sulfur protein 2) [48].

**Table 2:** miRNAs upregulated in dilated cardiomyopathy.

miRNAs	Quantitative Effect	Source	Notes
mi-RNA-155 miRNA-133a	Upregulated	Endomyocardial biopsies from patients with inflammatory DCM	MiRNA-133a has a role in the modulation of inflammation [57].
miR-146a	Upregulated	Exosome-specific microRNAs in plasma of patients with heart failure	It is induced in response to inflammation [59].
miRNA-30c	Upregulated	In vitro studies with transgenic mice	This dysregulation promotes a downregulation of OXPHOS complexes III and IV, which results in a coexistence of mitochondrial dysfunction [61].
miRNA-7	Upregulated	In vitro studies with transgenic mice	It promotes a reduction in NDUFA9, a component of NADH dehydrogenase complexes [62].
miRNA-194, miRNA-29a, miRNA 122, miRNA 423-5p	Upregulated	Exosomes from obese human plasma. It is correlated with cardiac dysfunctional parameters, including reduction in ejection fraction (EF) and increased levels of NT-proBNP	These miRNAs promote mitochondrial dysfunction through various mechanisms [63,64,65,66].
miRNA-143/145	Upregulated	Mouse cardiomyocytes	These microRNAs promote a reductive redox shift through several mechanisms of mitochondrial dysfunction [68].
miRNAs-221/222	Upregulated	Mouse cardiomyocytes	They determine a significative reduction in autophagy, by downregulating P27 and activating mTOR [69,70].
miRNA-22	Upregulated	Hearts explanted from DCM patients	It involves not only mTOR but also causes a decrease in PP2Cm, which catalyzes branched chain amino acids (BCAAs), determining inflammasome activation [71].
MiRNA-218-5p	Upregulated	Human iPSc differentiated cardiomyocytes	It activates TGF-β and promotes profibrotic effect [74].
MiRNA-208	Upregulated	Endomyocardial biopsy tissue in patients with DCM and controls	It is associated with alpha-myosin heavy chain (MHC) mRNA expression and correlates with poor clinical outcomes [73].
miRNA-21	Upregulated	Animal models	The overexpression of profibrotic genes, contributing to increased collagen production and myocardial fibrosis [75,76,77].
miRNA-33a	Upregulated	Human cardiac tissue samples with DCM	It is involved in cardiac remodeling by preserving lipid raft cholesterol content in fibroblasts; its upregulation is associated with improved hemodynamic parameters [78].
miR-3135b, miR-3908 and miR-5571-5p, exo-miR-92b-5p	Upregulated	Plasma samples from DCM patients and healthy controls	They could be used as DCM diagnostic biomarkers. miR-5571-5p also correlates with the severity of NYHA classification [81,82].
miRNA 15-b-5p miRNA-106a-5p	Upregulated	Plasma samples from DCM patients and healthy controls	The expression levels of these miRNAs may be helpful in distinguishing between ischemic and non-ischemic dilated cardiomyopathy [83].
miRNA 185	Upregulated	Plasma samples from DCM patients and healthy controls	High miRNA 185 levels could be associated with a better prognosis in DCM patients by repressing B cell function [82,83].
miRNA 338-3p	Upregulated	Left ventricular samples of heart failure mice	It suppresses cardiac fibroblasts activation and their differentiation into myofibroblasts [82,83].

**Table 3:** miRNAs downregulated in dilated cardiomyopathy.

miRNAs	Quantitative Effect	Source	Notes
miRNA-1, miRNA-23, miRNA-142-5p, miRNA-155, miRNA-193 and miRNA-195	Downregulated	Cardiac biopsies of patients with virus-negative inflammatory DCM	These miRNAs have been identified as part of a cardiac inflammatory process and thus as a possible therapeutic target [87].
mi-RNA 451	Downregulated	CD4+ T cells of DCM patients	Target the transcription factor Myc with the activation of CD4+ T cells [58].
miRNA 181b	Downregulated	Plasma of patients with heart failure	This miRNA is inhibited by IL-1 (IL-1β), interleukin-6 (IL-6) and TNF-α, markers of inflammation [60].
MiRNA-448-3p	Downregulated	Hearts of mdx mice, an animal model of DMD	The dysregulation of these miRNAs causes an increase in the NCF1 gene and p47phox protein; determining an increase in ROS and therefore tissue damage [67].
miRNA-1, miRNA-29c, miRNA-30c, miRNA-30d, miRNA-149, miRNA-486 and miRNA-499	Downregulated	Validated murine phospholambam mutant model of DCM	The downregulation of these miRNAs promotes apoptosis of myocardial cells [72].
miRNA-26, miRNA-29, miRNA-30	Downregulated	Animal models	The overexpression of profibrotic genes contributes to increased collagen production and myocardial fibrosis [75,76,77].
miRNA-142-3p and miRNA-548	Downregulated	Peripheral blood mononuclear cells (PBMCs) from patients with heart failure and DCM	The dysregulation of these miRNAs has been documented in patients with DCM and heart failure compared to healthy controls [79,80].
miRNA let-7i	Downregulated	Endomyocardial biopsy tissues from DCM patients and healthy controls	It regulates the expression of Toll-like receptor (TLR) 4, determining worse clinical outcomes in DCM patients [84].

**Table 4:** Lnc-RNAs in hypertrophic cardiomyopathy.

LncRNAs	Quantitative Effect	Source	Notes
LncRNA ADAMTS9-AS1	Upregulated	Serum of HOCM patients	It regulates cardiomyocyte hypertrophy and decreases levels of ANP and BNP [95].
LncRNA MIAT	Upregulated	Patients with HCM suffering from fibrosis and patients with HCM free of fibrosis	It is implicated in the control of fibrosis in HCM, through the modulation of miRNA29a-3p expression [96].
LncRN Ah19	Upregulated	Heart failure tissues	It promotes cardiac hypertrophy by targeting CaMKIIδ through miR-675 [97].
LncRNA CAIF	Downregulated	Myocardial tissues and serum of patients with end-stage cardiomyopathy and healthy controls	Patients with HCM end-stage had lower levels of lncRNA CAIF compared to controls [98].
LncRNA–mRNA pair (MIR210HG–BPIFC)	Downregulated	AC16 cells	It promotes the infiltration of naïve CD4+ T cells and CD8+ T cells in the myocardial cells of HCM patients [99].

**Table 5:** miRNAs upregulated in hypertrophic cardiomyopathy.

miRNAs	Quantitative Effect	Source	Notes
miRNA-27a, miRNA-29a, and miRNA-199a-5p	Upregulated	Plasma of HCM patients	Regulate cardiomyocyte hypertrophy and fibrosis [100].
miRNA-18a-5p, miRNA-146a-5p, miRNA-30d-5p, miRNA-17-5p, miRNA-200a-3p, miRNA-19b-3p, miRNA-21-5p, miRNA-193-5p, miRNA-10b-5p, miRNA-15a-5p, miRNA-192-5p, miRNA-296-5p, miRNA-29a-3p, and miRNA-133a-3p	Upregulated	Plasma of HCM patients	miRNAs promote cardiac fibrosis by acting on several pathways, including the control of the TGF-β/CTGF signaling cascade, ECM proteins, fibroblasts, and epithelial-to-mesenchymal transition [102].
miRNA-181a-5p, miRNA-181c-5p, miRNA-193b-3p, miRNA-301a-3p, and miRNA-328-3p	Upregulated	Serum from HCM sarcomere variant carriers with and without a diagnosis of HCM and healthy controls	Their levels increased in patients with clinical and subclinical HCM with early phenotypic manifestations [103].
miRNA-1-3p, miRNA-23a-3p, miRNA-23b-3p, miRNA-24-3p, miRNA-29b-3p, miRNA-30d-5p, miRNA-125a-5p, miRNA-126-3p, miRNA-133a-3p, miRNA-143-3p, miRNA-145-5p, miRNA-193b-3p, miRNA-197-3p, miRNA-331-3p, miRNA-342-3p, miRNA-361-5p, miRNA-365-3p, miRNA-455-3p, miRNA-1975-3p, and miRNA-1978	Upregulated	Healthy and diseased human hearts	They can be involved in the prognosis of patients with HCM [104,105].
miRNA-184, miRNA-497, miRNA-204, miRNA-34b, and miRNA-222	Upregulated	Cardiac tissue of HCM patients	They are implicated in ventricular hypertrophy and the β-adrenergic pathway [106].
miRNA-499a-5p	Upregulated	Plasma of HCM patients	Its level was higher in carriers of pathogenic/likely pathogenic (P/LP) variants in MYH7 gene compared to controls [108].
miRNA-20	Upregulated	In vitro model of hypertrophic cardiomyocytes	It promotes cardiac hypertrophy by reducing the MFN2 expression [111].
miRNA-221	Upregulated	Plasma and myocardial tissue	In patients with HOCM, it is correlated with myocardial fibrosis and hypertrophy [112].
miRNA-1, miRNA-98, and miRNA-924	Upregulated	PBMCs	It may be useful for detecting HCM [113].
miRNA-124-3p	Upregulated	Transcriptomic profiling data of patients with HCM	It may have a role in the pathophysiology of HCM by regulating the RhoA signaling pathway [114].

**Table 6:** miRNAs downregulated in hypertrophic cardiomyopathy.

miRNAs	Quantitative Effect	Source	Notes
miRNA-199a-3p	Downregulated	Two mouse models of HCM	Its inhibition may promote beneficial therapeutic effects in patients with moderate HCM [101].
miRNA-10b	Downregulated	Cardiac tissue of HCM patients	It is implicated in ventricular hypertrophy and the β-adrenergic pathway [106].
miRNA-208b-3p	Downregulated	Blood of HCM patients	It is associated with cardiac hypertrophy through Wnt signaling pathway [107].
miRNA-451	Downregulated	Heart tissue from HCM patients; neonatal rat cardiomyocytes	In cardiac cells, it promotes autophagosome formation by targeting TSC1 [109].
miRNA-139-5p	Downregulated	Neonatal rat cardiomyocytes	It promotes cardiac hypertrophy by targeting the transcription factor C-JUN [110].

**Table 7:** miRNAs in arrhythmogenic cardiomyopathy.

miRNAs	Quantitative Effect	Source	Notes
miRNA-185-5p	Upregulated	Plasma samples from ACM patients and healthy controls	It is significantly upregulated compared to controls; therefore, it could be used as a diagnostic biomarker [122].
miRNA-21-3p, miRNA-21-5p, miRNA-34a-5p, miRNA-212-3p, miRNA-216a, miRNA-584-3p, miRNA-1251, miRNA-3621, miRNA-3674, miRNA-3692-3p, miRNA-4286, miRNA-4301	Upregulated	Heart tissue of transplant patients with ACM	The most severely dysregulated was MiRNA-21-5p, implicated in Wnt and Hippo signaling pathways and involved in fibrosis and fibrofatty infiltration [124,125].
miRNA-29b-3p	Upregulated	ACM cardiac stromal cells	It contributes to ACM pathogenesis and phenotype maintenance [126].
miRNA-144-3p, 145-5p, 185-5p, and 494	Upregulated	Plasma samples from ACM patients and patients with idiopathic ventricular controls	These miRNAs are upregulated in ACM patients with VT. MiR-494 is associated with the recurrence of VT after ablation [128].
miRNA-320a	Downregulated	Plasma samples from ACM patients and healthy controls	It regulates cardiomyocyte survival and the WNT pathway by directly targeting β-catenin [121].
miRNA-135b, miRNA-138-5p, miRNA-193b-3p, miRNA-302b-3p, miRNA-302c-3p, miRNA-338-3p, miRNA-451a, miRNA-491-3p, miRNA-575, miRNA-3529-5P, miRNA-4254, miRNA-4643	Downregulated	Heart tissue of transplant patients with ACM	The most severely dysregulated was miRNA135b, implicated in Wnt and Hippo signaling pathways and involved in fibrosis and fibrofatty infiltration [124,125].
